# Structural and molecular changes in the rat myocardium following perfluoroctane sulfonate (PFOS) exposure are mitigated by quercetin via modulating HSP 70 and SERCA 2

**DOI:** 10.1007/s10735-023-10134-9

**Published:** 2023-06-27

**Authors:** Dalia A. Mandour, Manal M. Morsy, Amal Fawzy, Noura Mostafa Mohamed, Marwa M. Ahmad

**Affiliations:** 1 Department of Human Anatomy and Embryology, Faculty of Medicine, Zagazig University, Zagazig, Egypt; 2 Department of Biochemistry, Faculty of Medicine, Zagazig University, Zagazig, Egypt

**Keywords:** PFOS, Myocardium, Immunohistochemistry, Gene expression, Quercetin, Rats

## Abstract

Perfluorooctane sulfonate (PFOS) is a man-made fluorinated compound employed in a variety of industrial and civilian applications. Due to its long elimination half-life and promotion of oxidative stress and inflammation, it is one of the most abundant organic contaminants. The present study was designed to determine the cytotoxic effect of PFOS on adult male rat cardiac tissue and to assess the cardioprotective role of the flavonoid quercetin (Que), which possesses antioxidant, anti-inflammatory, and anti-apoptotic properties. Twenty-four adult male Sprague–Dawley rats were randomly divided into four equal groups: Group I (Control). Group II (Que) received Que (75 mg/kg/day for 4 weeks) by oral gavage. Group III (PFOS group): supplemented orally with PFOS (20 mg/kg/day for 4 weeks) and Group IV (PF OS/Que). The rat heart was processed for histological, immunohistochemical, and gene expression studies. The PFOS group showed histological alterations in the myocardium that were partially reversed by the administration of Que. The inflammatory biomarkers (TNF, IL-6, and IL-1), lipid profile, TSH, MDA, and serum cardiac enzymes (LDH and CK-MB) were all altered. These findings collectively suggest that PFOS had adverse effects on the cardiac muscle structure, and these effects were alleviated by quercetin, which is a promising cardioprotective flavonoid.

## Introduction

Perfluorinated compounds (PFCs) are a group of man-made chemicals used in a wide range of industrial applications and many consumer products due to their stable and strong lipophobic and hydrophobic properties. (Giesy and Kannan 2001). PFCs are used in stain and grease repellents, non-stick coatings, aqueous firefighting foams, paper plates, carpet cleaning solutions, some adhesives, pharmaceuticals, electronics, cleaning products, and paints. (Buck et al. 2011; Cui et al. 2009b; Kissa 2001). Moreover**,** PFCs have been widely used in various commercial applications, including cosmetics, lubricants, water and oil surfactants, pesticides, textile coatings, fast food packaging, polishes, and fire-retarding (Chang et al. 2016; Dong et al. 2012). PFCs are environmentally persistent and bioaccumulative contaminants that have been ubiquitously found in the environment. They reach the water, atmosphere, soil, sludge, and other environmental media, causing widespread pollution (Cui et al. 2009b).

Consequently, they are transferred to animals and humans from the water supply and food chain and, in turn, bioaccumulated in various tissues of the body, hampering a potential health risk (Cui et al. 2009b; Houde et al. 2006; Kannan et al. 2004). PFCs have been detected in a variety of media and biota, including public water supplies, human serum, and breast milk (Sunderland et al. 2019).

Human exposures to PFCs may occur directly by touching PFC-containing consumer products, followed by hand-to-mouth contact (Guo et al. 2009). Moreover, food becomes contaminated directly from food packaging coated with grease and water-repellent coatings.(e.g., fast food containers, microwave popcorn bags) (Begley et al. 2005) or by ingestion of PFCs bioaccumulated in animal or plant-based foods (Tittlemier et al. 2007) or drinking contaminated water (Ericson et al. 2008). Exposures may occur indirectly through exposure to indoor air (Shoeib et al. 2004) and dust (Kubwabo et al. 2005).

Perfluorooctane sulfonate (PFOS) is a prominent member of the PFCs and is defined as one of the environmentally persistent pollutants that are not susceptible to degradation and are slowly eliminated by the human body (Ping et al. 2007). PFOS is easily absorbed through the gut, is poorly eliminated, and is not metabolized. Unlike most persistent chemicals, they bind to proteins rather than fats (lipids) and are distributed mainly to the blood, serum, kidneys, and liver (Lau et al. 2007). Many studies have indicated that PFOS has many adverse health concerns, including hepatotoxicity, developmental toxicity, reproductive toxicity, neurotoxicity, endocrine disruption, immune injury, and carcinogenesis (Cui et al. 2009a; Klaunig et al. 2012; Lau et al. 2007; Olsen et al. 2009).

In addition, epidemiological investigations in the general population have revealed that elevated concentrations of serum PFOS are associated with thyroid disease (Melzer et al. 2010).

Due to public health and environmental concerns, the International Agency for Research on Cancer (IARC) classified perfluorooctanoic acid (PFOA) and perfluorooctane sulfonic acid (PFOS) as possible carcinogens (2B group) in humans. (Group 2016).

Heart disease is one of the most important causes of high mortality in the world, and exposure to environmental pollutants is evidenced as a risk factor for cardiovascular diseases. CVD (Mastin 2005; Roth et al. 2015). Many studies reported that PFCs exposure can be added to the risk factors of heart disease as it affects body weight, glucose homeostasis, and insulin resistance (Geiger et al. 2014; Nelson et al. 2010). Furthermore, several studies have found favourable relationships between blood PFOA and PFOS and lipid profiles, and the link between these chemicals and dyslipidemia is well known as a CVD risk factor (Liu et al. 2018).

HSPs, which include several families of cytoprotective proteins, are responsible for molecular chaperone activity. The expression of molecular chaperones, which are important components of cardiomyocytes, was low during normal cardiac function but increased during cardiac stress. The most abundant is HSP70, which has been studied for its chaperone function in general as well as its role in the cardiovascular system. Hsp70 levels and distribution occurred during the stress process, which correlated with the severity of myocardial injury (Agashe and Hartl 2000).

Thyroid hormone (TH) is important for growth, development, and metabolism in practically every organ (Yen 2001). Exposure to PFOS reduces circulating TH levels in both monkeys and rodents (Curran et al. 2008). PFOS was found to reduce the activity of thyroid peroxidase (TPO), the key enzyme responsible for the synthesis of thyroid hormones (Ballesteros et al. 2017). There are many cardiac genes considered targets of transcriptional stimulation by TH.

Quercetin (Que) is a type of natural flavonoid found in a variety of foods, including onions, apples, and tea (Hertog et al. 1993; Morand et al. 1998). Que is crucial for the prevention and treatment of type 2 diabetes, obesity, and heart disease (Xiao et al. 2017). Many researchers have recently become interested in quercetin, which has been shown to have antioxidant, anti-inflammatory, anti-clotting, and vasodilatory characteristics (Rivera et al. 2008).

Despite numerous investigations, the underlying mechanisms of PFOS toxicity on heart tissue remain unknown; hence, the current study aims to assess the direct apoptotic, oxidative, and inflammatory impacts of PFOS on heart tissue, as well as its indirect effects via alteration of thyroid hormone levels. Alterations in the myocardial tissue resulted from PFOS exposure, as assessed biochemically and histologically.

## Material and methods

### Chemicals and animals

Perfluorooctane sulphonate was provided as a white powder of 98% purity with a CAS number of 2795-39-3 (Sigma Aldrich Chemical Co., St. Louis, MO, USA). It was dissolved in 0.5% Tween-20 in deionized water, and quercetin was provided as a solid, purity 95%, with a CAS number of 117-39-5 (Sigma Aldrich Chemical Co., St. Louis, MO, USA). It was dissolved in distilled water.

Adult male Sprague–Dawley rats weighing approximately 190–230 grammes were used in this study. They were purchased from the animal house, Faculty of Medicine, Zagazig University, and allowed to acclimate for one week prior to the initiation of the experiment. The rats were housed at room temperature with a 12-h dark/light cycle and were fed standard rat chow and tap water ad libitum. This study was conducted in strict accordance with the recommendations of the National Institutes of Health’s Guide for the Care and Use of Laboratory Animals. The research protocol with animal experimentation was approved by the Institutional Committee for Ethical Care and Use of Laboratory Animals, Faculty of Medicine, Zagazig University, Egypt (a reference number: ZU-IACUC/3/F/206/2021).

### Experimental design

In this study, twenty-four rats were utilized, and they were randomly separated into four groups of equal size, each with six rats: The Control Group (Group I): Group I received only a regular diet and distilled water for 4 weeks; Group II received oral gavage of Que supplementation (75 mg/kg/day for 4 weeks) (Srinivasan et al. 2018), Group III received oral gavage of PFOS supplementation (20 mg/kg/day for 4 weeks) (Cui et al. 2009a) and Group IV received oral gavage of PFOS (20 mg/kg/day) and oral Que supplementation (75 mg/kg/day) simultaneously for 4 weeks.

### Blood and tissue sampling

The rats were fasted overnight after the treatment phase ended on the 28th day of the experiment. They were sedated with a single intraperitoneal dose of sodium pentobarbital (50 mg/kg BW) the next morning, and blood samples were taken from the retro-orbital venous sinuses and left to clot at room temperature in non-heparinized tubes before being centrifuged at 1000 × g for 20 min. The serum samples were kept at a temperature of 20 °C until biochemical analysis. Thereafter, a thoracotomy was performed to gain access to the heart that was dissected and excised outside the body; each isolated heart was divided longitudinally into two halves. One half was fixed in 10% neutral-buffered formalin for histological studies, and the second half was immediately preserved at − 80 °C for further homogenization to serve for biochemical and gene expression profile analysis.

### Histological studies

#### Light microscopic study

The formalin-fixed heart specimens were dehydrated in ascending grades of ethanol and cleared in xylene, then embedded in paraffin blocks from which 5 m-thick sections were cut and stained with hematoxylin and eosin (H&E) stain (for routine histological examination) (Bancroft and Layton 2013) and Van Gieson’s stain (for assessing the amount of the collagen in between the cardiac muscle fibers) (Benard et al. 2017)**.** The stained H&E and Van Gieson’s sections were examined under a light microscope (Leica DM 500, Microsystems, AG, Heerbrugg, CH-9435, Switzerland) and photographed with a digital camera (Leica ICC50 W) coupled to that microscope in the Department of Human Anatomy and Embryology, Faculty of Medicine, Zagazig University, Egypt.

### Immunohistochemical (IHC) study

Longitudinal heart sections of 4 m thick were obtained from the paraffin blocks of the formalin-fixed heart specimens and mounted on positively charged slides, then deparaffinized in xylene, rehydrated in descending grades of ethanol, incubated with hydrogen peroxide to block the endogenous peroxidase activity, and subjected to antigen retrieval and blocking of non-specific antigens. Afterwards, the slides were incubated with PBS-diluted primary antibodies against Connexin 43 (CX43; rabbit polyclonal; dilution 1:2000; Sigma-Aldrich, Oakville, CA) and against Heat Shock Protein 70 (HSP70; mouse monoclonal; dilution 1:200; Thermo Fisher Scientific). The slides were incubated overnight at 4 °C, then washed three times with phosphate buffer solution (PBS). Afterwards, the slides were incubated with their corresponding biotin-labelled secondary antibodies, followed by conjugation with streptavidin peroxidase. Diaminobenzidine (DAB, Sigma-Aldrich Chemical Co., St. Louis, MO, USA), as a chromogen solution, was added to the slides to visualise the antigen–antibody-peroxidase reaction. Finally, the sections were counterstained with Mayer’s hematoxylin. The positive immunoreactivity was indicated by brown staining of the antigen sites. Some slides were prepared without incubation with primary antibodies to serve as controls for non-specific staining (Ramos-Vara et al. 2008). All immunostained sections were examined and photographed under the light microscope.

### Histomorphometric analysis

The mean area percentage of collagen fibres in the Van Gibson’s-stained sections and the mean area percentage of CX43 and HSP70 immunoreactivity were measured in six non-overlapped high-power fields (× 400) from each group. These area percentages were quantitatively estimated using the ImageJ software analyzer computer system (NHI, Wayne Rasband, Bethesda, Maryland, USA). Then, the obtained data were presented as mean-SD and statistically analyzed.

Biochemical parameters: The separated sera were used for determination of the following: level of cardiac enzymes, thyroid hormones, and lipid profile.

Serum LDH and serum CK-MB were determined using the colorimetric assay kits (Stanbio Laboratory, Boerne, Texas, USA). Serum levels of T3, T4, and TSH were determined using the ELISA assay kits, following the manufacturer's instructions. The total plasma cholesterol was measured by quantitative, enzymatic, and colorimetric determination of total and HDL cholesterol in serum (Cholesterol Assay Kit, HDL Cholesterol Assay Kit, HDL (ab65390). determination of serum triglyceride (TG) levels. The plasma triglyceride level was measured by quantitative, enzymatic, and colorimetric determination of triglycerides in serum (Triglyceride Assay Kit, Quantification (AB65336)). Serum low-density lipoprotein cholesterol (LDL-C) was calculated as follows: LDL = TC-HDL-TG/5 according to Friedewald et al. (1972). VLDL-c was measured using Friedewald et al.’s (1972) formula.

### Determination of cardiac antioxidant activity and lipid peroxidation

To investigate the cardiac oxidative damage, the levels of MDA, a marker of lipid peroxidation were measured in the heart using a commercially available ELISA kit (MDA Elabscience® ELISA Kit Catalog No. E-EL-0060, USA) according to the manufacturer’s instructions. The CAT activity was assayed using a commercially available assay kit (CAT Elabscience® ELISA Kit Catalog no. E-EL-R2456, USA) according to the manufacturer’s instructions. The CAT activity was expressed as U/g for cardiac tissue homogenates. The SOD activity was measured in accordance with the protocol supplied by commercially available SOD assay kit (SOD Elabscience® ELISA Kit Catalog no. E-EL-R1424, USA), and the SOD activity was expressed as U/g protein for cardiac tissue homogenates.

### Determination of tumor necrosis factor (TNF-a), Interleukin-6 (IL-6), interleukin-1 beta (IL-1ß) levels in cardiac tissue

Cardiac tissue homogenates were prepared in phosphate buffer and centrifuged. Supernatants were collected, and TNF-, IL-6, and IL-1ß levels were assessed using the ELISA method provided by the manufacturers (Raybiotech Co., Peachtree Corners, United States); the values were expressed as pg/ml.

### Gene expression analysis

#### Sample preparation and RNA isolation

Total RNA was isolated from frozen myocardial tissue according to the RNA isolation kit (Gentra, Minneapolis, MN 55441, USA), following the manufacturer’s protocol. The purity and integrity of total RNA were monitored by the absorbance of ultraviolet light spectrophotometrically at 260 and 280 nm.

### Reverse transcription and complementary DNA (cDNA) synthesis

For the synthesis of complementary DNA (cDNA), the extracted RNA was reverse transcribed by the QuantiTect SYBR Green reverse transcription (RT-PCR) kit (Qiagen; catalogue no. 204243) as recommended by the manufacturer. The cDNA was stored at 20 °C until the time of analysis.

### Measurement of p53, Bax, Bcl-2, caspase-3, and SERCA2a mRNA expression by real time polymerase chain reaction

Expression levels were determined by real-time polymerase chain reaction using the Step One TM System (Applied Biosystems). The glyceraldehyde-3-phosphate dehydrogenase gene (G3PDH) was used as an internal control. Primers’ sequences for genes were reported in (Table 1). The PCR was performed in 25 mL containing 12.5 mL of QuantiFast SYBR Green (Cat. No. 204141), PCR Master Mix, 1 mM of each primer (Invitrogen, USA), and 2 mL of cDNA. For p53, Bax, Bcl-2, and caspase-3, the thermal cycling status was an initial denaturation at 95 °C for 2 min followed by 40 cycles of denaturation at 94 °C for 30 s, annealing at 52 °C for 60 s, and elongation at 72 °C for 2 s for 40 cycles. after a final incubation at 72 °C for 7 min. For SERCA2a, the denaturation was done at 95 °C for 20 s, the annealing at 60 °C for 30 s, and the elongation at 72 °C for 2 s for 40 cycles. The expression of all genes was reported as the D-cycle threshold (DCt) value. All kits were supplied by QIAGEN (Valencia, CA).

**Table 1:** Primers of p53, Bcl-2, caspase-3 and SERCA2a

Gene	Sense and antisense
p53	5′-AAGGAAATTTGCGTGTGGAG-3′ 5′-TTCTGACGCACACCTATTGC-3′
Bcl-2	5′-TTGTGGCCTTCTTTGAGTTCG-3′ 5′-TACTGCTTTAGTGAACCTTTT-3′
Bax	F: 5′AGGGTGGCTGGGAAGGC3′ R: 5′TGAGCGAGGCGGTGAGG3′
Caspase-3	5′-ATGGACAACAACGAAACCTCCGTG-3′ 5′-CCACTCCCAGTCATTCCTTTAGTG-3′
SERCA2a	5′-CGAAAACCAGTCCTTGCTGAGGAT-3′ 5′-TACTCCAGTATTGGCATGCCGAGA-3′

### Statistical analysis

The data from the biochemical investigation, as well as the mean area percent of collagen fibers and area percent of both CX43 and HSP70 immunostaining, were analyzed using the Statistical Package for Social Science application. (SPSS, version 19, Inc., Chicago, IL, USA). These data were expressed as mean SD. A one-way ANOVA test was used to determine the statistical significance among the different groups (Petrie and Sabin 2019). The results were considered statistically significant when their P values were less than 0.05.

## Results

### Histological results

#### Results of hematoxylin and eosin (H&E) staining

**Fig. 1:**
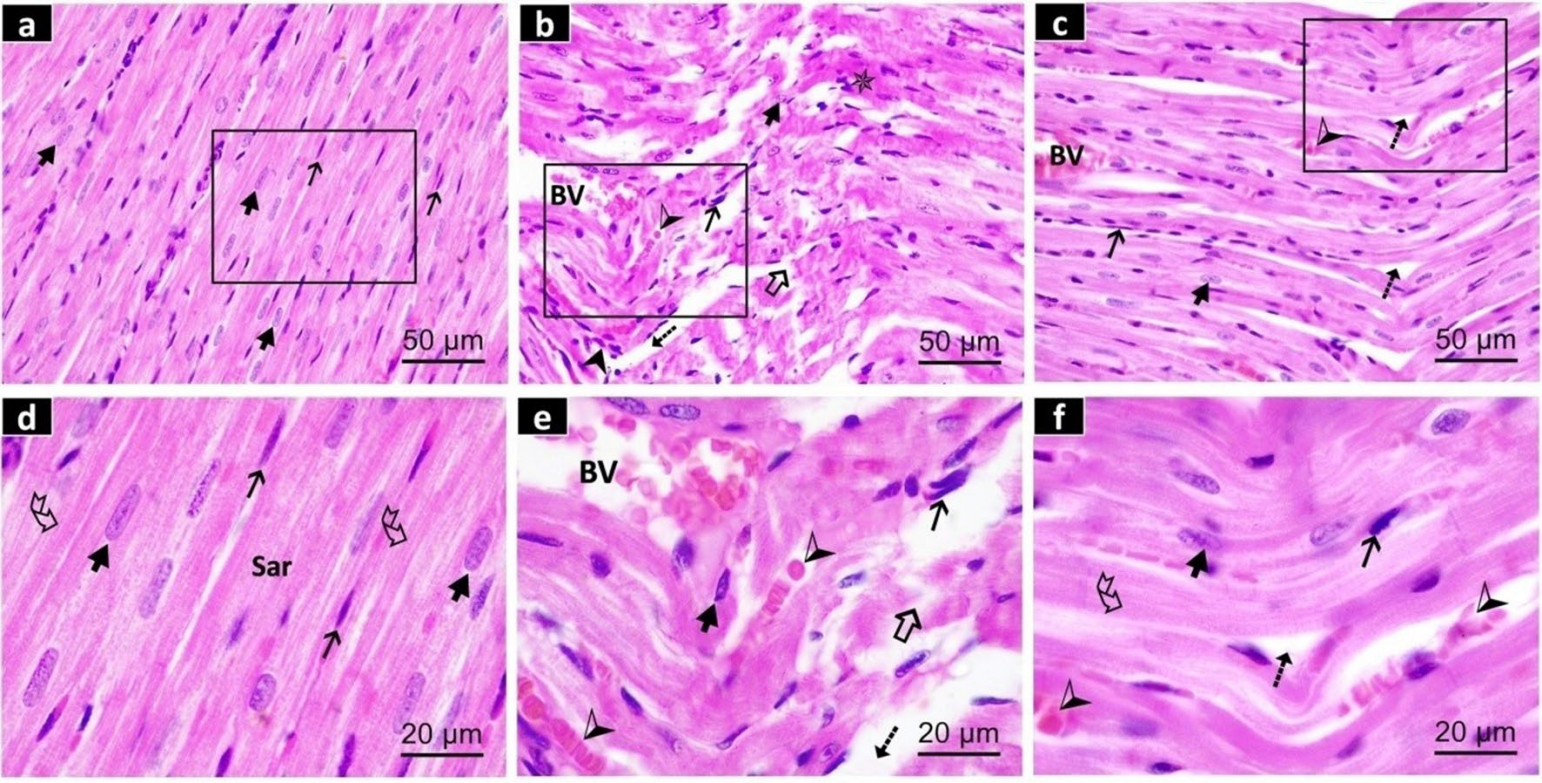
Photomicrographs of H&E-stained sections of the rat ventricular myocardium in all experimental groups. **a**, **d** The control group exhibits normal cardiac muscle fibers with oval nuclei () in acidophilic sarcoplasm (**sar**) having a normal pattern of cross-striations (). The connective tissue between the cardiomyocytes discloses dark elongated nuclei of fibroblast ().** b**, **e** The PFOS group displays irregularly-arranged cardiac muscle fibers having pyknotic nuclei () and deeply stained areas () of the sarcoplasm without cross-striations, in addition, some muscle fibers are fragmented and discontinuous (). Wide intercellular spaces () with many intervening cardiac fibroblasts (), dilated congested blood vessels **(BV)** with extravasated RBCs () and inflammatory cellular infiltration () are observed. **c**, **f** The PFOS/Que group discloses some slightly arranged cardiac muscle fibers with oval vesicular nuclei () and preserved cross-striations () of the sarcoplasm. Other muscle fibers are distorted with intercellular spaces () between them, together with a moderate amount of cardiac fibroblasts (), extravasated RBCs (), besides slightly dilatated congested blood vessels **(BV)**. Scale bar of a, b, c = 50 µm (H&E × 400) and scale bar of d, e, f = 20 µm (H&E staining × 1000)

Longitudinal sections in the ventricular myocardium of groups I (control group) and II (que group) exhibited a nearly similar histological architecture of longitudinally arranged cardiac muscle fibers (cardiomyocytes) that appeared branched. The cardiomyocytes revealed normal oval, centrally located nuclei in an acidophilic sarcoplasm that displayed a normal pattern of cross-striations. Additionally, the connective tissue of the endomysium among the cardiac muscle fibres revealed spindle-shaped, darkly stained fibroblasts (Fig. 1a, d). The group III (PFOS group) displayed irregularly arranged cardiomyocytes with small, darkly stained pyknotic nuclei and deeply stained homogenous areas of the sarcoplasm that lost their cross-striations (coagulative necrosis). Some muscle fibres were fragmented, disrupted, and devoid of nuclei. Wide intercellular spaces among the cardiomyocytes entangled many fibroblasts, dilated congested blood vessels, extravasated RBCs, and inflammatory cellular infiltrates (Fig. 1b, e). The group IV (PFOS/Que group) revealed somewhat apparently arranged cardiac muscle fibers with oval vesicular nuclei and preserved cross-striations of the sarcoplasm. A significant number of cardiac fibroblasts, extravasated RBCs, inflammatory cells, and slightly dilated congested blood vessels separated some muscle fibers, making them wavy. (Fig. 1c, f).

## Results of Van Gieson’s staining

**Fig. 2:**
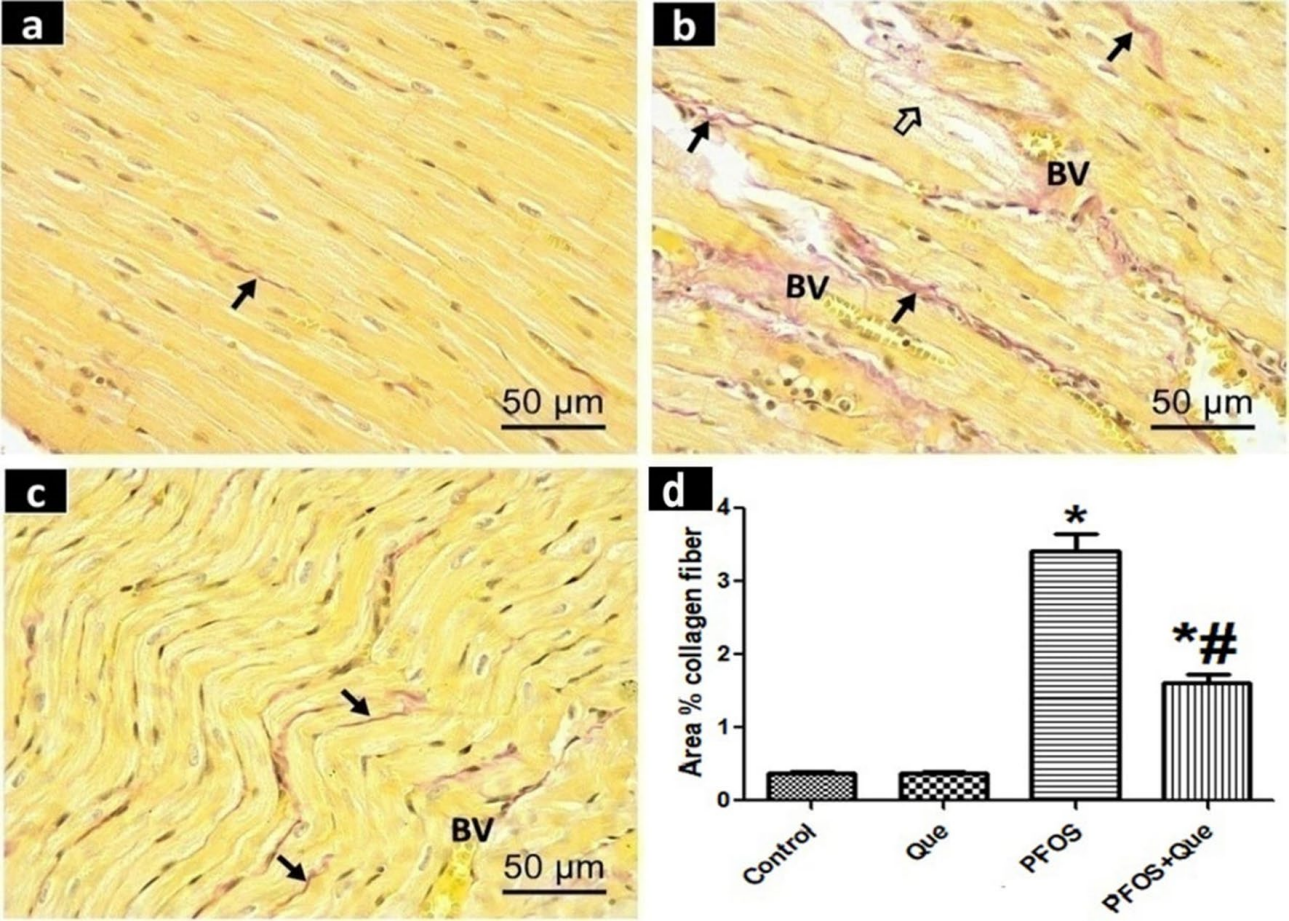
Photomicrographs of Van Gieson-stained sections of the rat ventricular myocardium of all experimental groups. **a** The control group reveals delicate red-stained collagen fibers () between the cardiomyocytes. **b** The PFOS group displays a marked increase in the amount of the interstitial red-stained collagen fibers () mainly close to the damaged myocardial fibers () and around the blood vessels **(B.V)**. **c** The PFOS/Que group shows little amount of collagen fibers () in the interstitium. Scale bar = 50 µm (Van Gieson’s staining × 400). **d** A bar graph showing the area % of collagen fibers in Van Gieson-stained sections. Values are expressed as Mean ± SD. Statistical analysis was carried out using One-way ANOVA followed by Tukey′s test. *Significant p value when compared to the control group. # Significant p value when compared to PFOS group

The ventricular myocardium of the control group exhibited a resemblance to the que group upon Van Gieson's staining, where a few fine, red-stained collagen fibers appeared in between the cardiac muscle fibers (Fig. 2a). The number of interstitial, red-stained collagen fibers in the PFOS group was much higher, particularly in areas of damaged heart muscle fibers and around blood vessels (Fig. 2b). The PFOS/Que group revealed an apparent decrease in the red-stained collagen fibers in the interstitium compared with the PFOS group. (Fig. 2c).

## Results of immunohistochemical staining of connexin 43 (CX43)

**Fig. 3:**
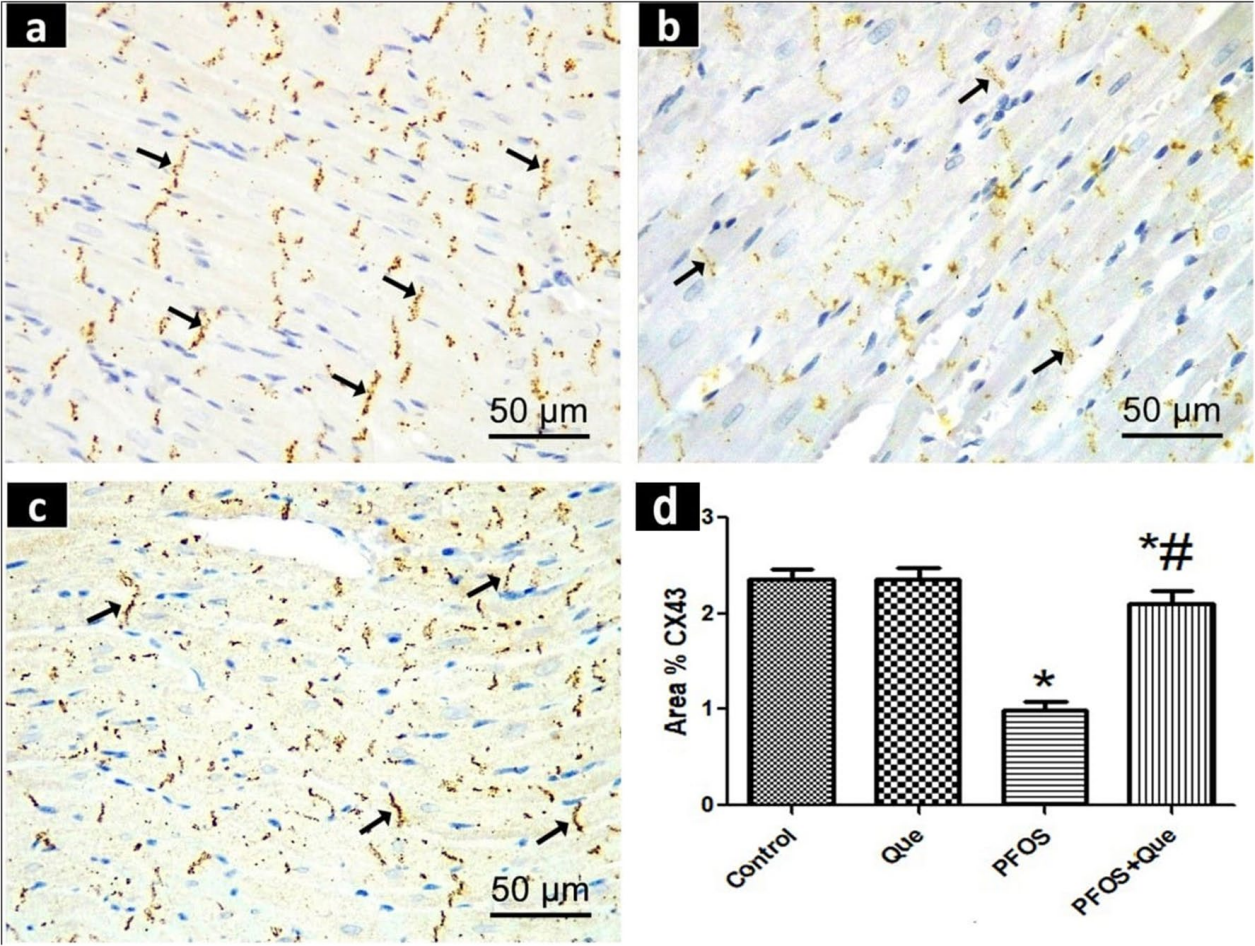
Photomicrographs of CX43-immunostained sections of the rat ventricular myocardium of all experimental groups. **a** The control group reveals extensive expression of CX43 in the intercalated discs (). **b** The PFOS group showed a weak CX43 expression in disrupted intercalated discs (). **c** The PFOS/Que group demonstrates a modest CX43 expression. Scale bar = 50 µm (CX43 immunostaining × 400).** d** A bar graph showing the area % of CX43 immunoreactivity. Values are expressed as Mean ± SD. Statistical analysis was carried out using One-way ANOVA followed by Tukey′s test. *Significant p value when compared to the control group. # Significant p value when compared to PFOS group

CX43 was highly expressed in the intercalated discs of the Control and Que groups, which showed dense, brown-stained cross bands that held the ends of cardiac muscle fibers together (Fig. 3a). The PFOS group revealed a weak CX43 expression in disrupted intercalated discs that emerged as faint brown cross bands (Fig. 3b). The PFOS/Que group displayed a moderate expression of CX43 in apparently preserved intercalated discs (Fig. 3c).

## Results of immunohistochemical staining of heat shock protein 70 (HSP70)

**Fig. 4:**
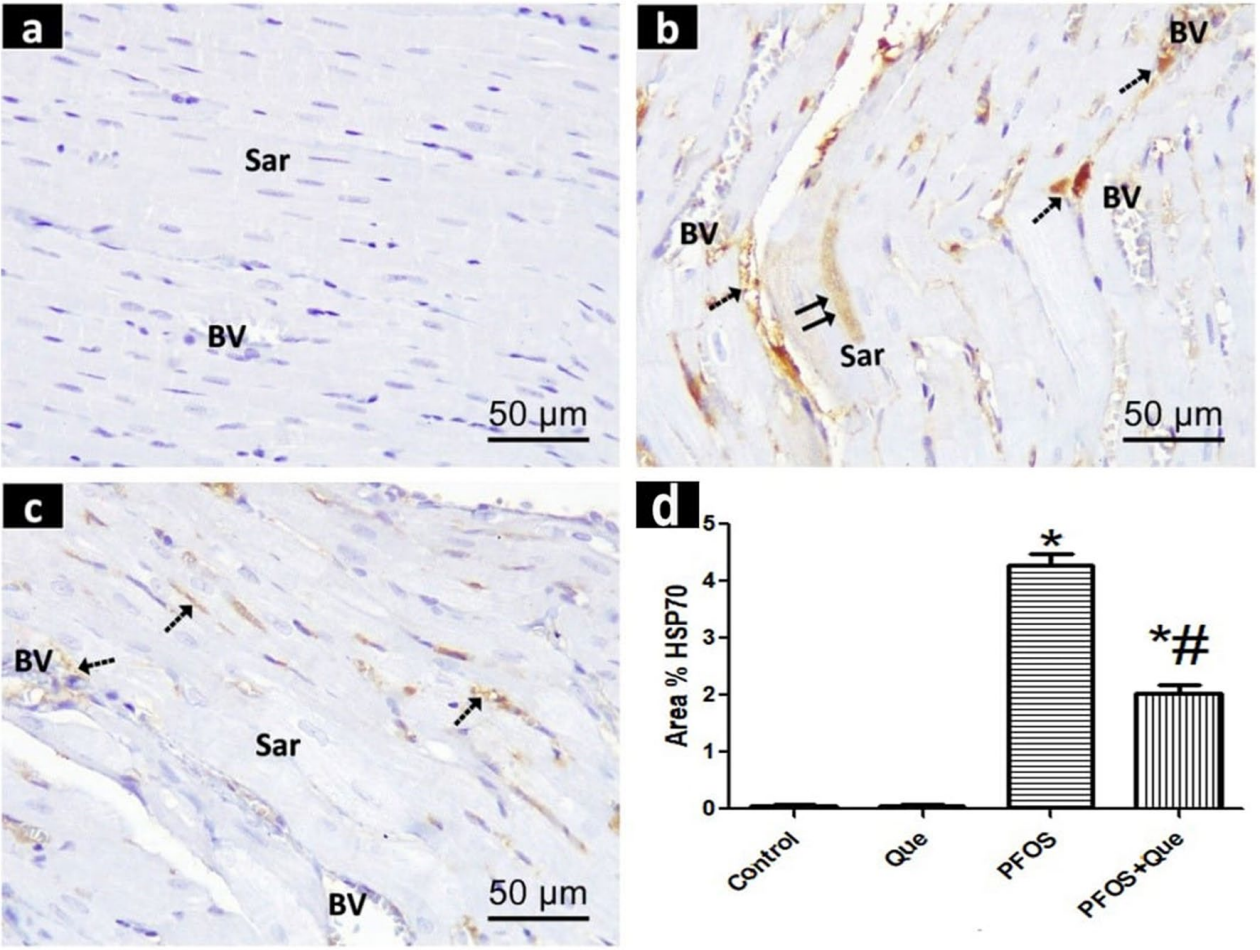
Photomicrographs of HSP70-immunostained sections of the rat ventricular myocardium of all experimental groups. **a** The control group reveals no expression of HSP70 in the blood vessels **(BV)** or in the sarcoplasm **(sar**) of the cardiomyocytes. **b** The PFOS group exhibits a positive expression of HSP70 in **BV**, in the perivascular compartments () between the muscle fibers and also in the** sar** (). **c** The PFOS/Que group demonstrates a weak expression of HSP70 in** BV** and perivascular compartments (), while a negative HSP70 immunoexpression in the** sar**. Scale bar = 50 µm (HSP70 immunostaining × 400). **d** A bar graph showing the area % of HSP70 immunoreactivity. Values are expressed as Mean ± SD. Statistical analysis was carried out using One-way ANOVA followed by Tukey′s test. *Significant p value when compared to the control group. # Significant p value when compared to PFOS group

*The Control* and *Que groups* exhibited negative HSP70 immunoreactivity in the myocardial sections (Fig. 4a). HSP70 immunoreactivity was found in tiny capillaries, perivascular compartments, and the sarcoplasm of heart muscle fibers in the PFOS group* (*Fig. 4b*). The PFOS/Que group* displayed a weak expression of HSP70 in the small capillaries and perivascular connective tissue, while it was negatively expressed in the sarcoplasm of the cardiac muscle fibers (Fig. 4c).

## Results of histomorphometry

PFOS group displayed a significant increase (P < 0.0001) in the mean area percentage (%) of collagen fibers compared with the control group. PFOS/Que group exhibited a significant decrease (P < 0.0001) in this mean area % compared to the PFOS group (Fig. 2d).

When compared to the control group, the mean area percent of CX43 immunoexpression in the PFOS group was statistically significant (P < 0.0001). This mean area percent increased significantly (P < 0.0001) in the PFOS/Que group compared to the PFOS group. (Fig. 3d).

In comparison to the control and Que groups, the PFOS group showed a substantial increase (P < 0.0001) in the area percent of HSP70 immunoexpression. When compared to the PFOS group, the PFOS/Que group showed a substantial decrease (P < 0.0001) in this area (Fig. 4d).

## Biochemical results

### Serum cardiac enzymes levels in the different studied groups

PFOS group showed a significant increase of cardiac enzymes (LDH and CK-MB) compared to controls (P < 0.05). The (PFOS + Que) treated group showed a significant decrease of LDH and CK-MB levels compared to the PFOS -exposed group (p < 0.001), while the cardiac enzymes still significantly increased compared to the control groups (Table 2).

**Table 2:** Serum cardiac enzymes in all studied groups

	Group I (control group)	Group II (Que group)	Group III (PFOS group)	Group IV (PFOS + Que group)
LDH (IU/L)	228 ± 6.4	230 ± 6.9	261 ± 5.2^a,b^	240 ± 4.3^a,b,c^
CK-MB(IU/L)	16 ± 1.2	17 ± 1.6	24 ± 1.0^a,b^	21.4 ± 0.4^a,b,c^

^a^Significant compared to group I (p < 0.05)

^b^Significant compared to group II (p < 0.05)

^c^Significant compared to group III (PFOS group) (p < 0.05)

### Thyroid hormone in PFOS-exposed rats

PFOS-exposed animals show significantly lower levels of thyroid hormones when compared with the control group. The PFOS + Que-treated group had significantly greater total T4 and total T3 levels than the PFOS-exposed group, as shown in Table 3.

**Table 3:** Serum levels of T3, T4, and TSH in all studied groups

	Group I (control group)	Group II (Que group)	Group III (PFOS group)	Group IV (PFOS + Que group)
Total T3(µg/L))	0.04 ± 0.29	0.03 ± 0.28	0.06 ± 0.18^a,b^	0.05 ± 0.24
Total T4( µg/L)	40.9 ± 1.8	38.5 ± 1.6	32 ± 6.3^a^	36.4 ± 5.4
TSH (IU/L)	0.72 ± 0.30	1.32 ± 0.67	1.82 ± 0.27^a^	1.12 ± 0.34^c^

^a^Significant compared to group I (p < 0.05)

^b^Significant compared to group II group (p < 0.05)

^c^Significant compared to group III (PFOS group) (p < 0.05)

### Lipid profile changes in PFOS-exposed rats

There was a statistically significant rise in blood total cholesterol, LDL, and triglycerides in the PFOS-treated groups when compared to the control groups. The PFOS + Que-treated group had significantly lower serum total cholesterol, LDL, and triglyceride levels (p 0.001) than the PFOS-exposed group; however, these values were still significantly higher than the control group. The results are shown in Table 4.

**Table 4:** Lipid profile in different studied groups

Groups	Group I (control group)	Group II (Que group)	Group III (PFOS group)	Group IV (PFOS + Que group)
HDL (mg/dl)	52.1 ± 0.8	50 ± 4.3	38.3 ± 4.2^a,b^	48 ± 3.2^c^
LDL (mg/dl)	88.8 ± 1.4	86 ± 2.6	95 ± 4.5^a,b^	92 ± 2.3^c^
Triglycerides(mg/dl)	87.4 ± 3.1	85 ± 2.4	97 ± 4.2^a,b^	84 ± 3.1^c^
Cholesterol (mg/dl)	90.6 ± 5.2	88.7 ± 6.3	118.0 ± 5.1^a,b^	98.7 ± 6.3^c^

^a^Significant compared to group I (p < 0.05)

^b^Significant compared to group II (p < 0.05)

^c^Significant compared to group III (PFOS group) (p < 0.05)

### Cardiac antioxidant enzymes and lipid peroxidation changes in PFOS-exposed rats

In comparison to the controls, PFOS injection demonstrated a substantial drop in CAT and SOD, as well as a large increase in MDA (P < 0.001). We found a significant rise in CAT and SOD, as well as a decrease in MDA, in the (PFOS + Que) treated group compared to the PFOS-exposed group (p 0.001), even though these parameters were still significantly higher than controls (Tables 4 and 5).

**Table 5:** Cardiac Catalase (CAT), Superoxide dismutase (SOD), Malondialdehyde (MDA), in the different studied groups

	Group I (control group)	Group II (Que group)	Group III (PFOS group)	Group IV (PFOS + Que group)
CAT (U/g tissue)	62.3 ± 2.2	61.2 ± 2.4	32.1 ± 2.2^a,b^	57.2 ± 3.2^a,c^
SOD (U/g tissue)	51.815 ± 1.39	51.021 ± 3.02	30.335 ± 3.42^a,b^	43.263 ± 3.46^a,b,c^
MDA (nmol/g tissue)	29.38 ± 1.75	27 ± 1.5	93.30 ± 9.63^a,b^	69.2 ± 6.4^a,b,c^

^a^Significant compared to group I (p < 0.05)

^b^Significant compared to group II (p < 0.05)

^c^Significant compared to group III (PFOS group) (p < 0.05)

### Cardiac tissue levels of inflammatory biomarkers in PFOS-exposed rats

**Fig. 5:**
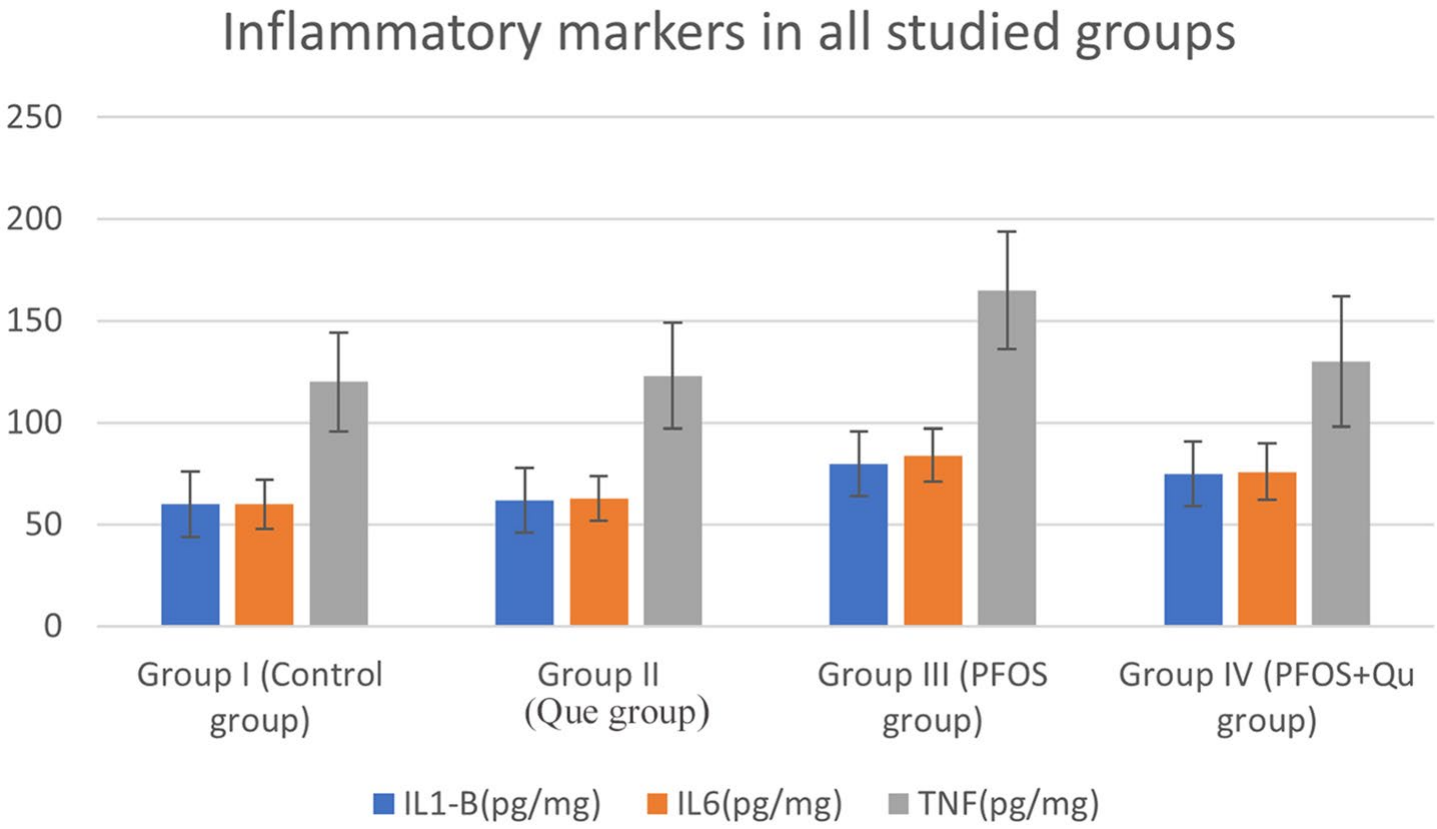
Bar charts representing the inflammatory markers in all studied groups

When compared to the control group, the levels of tumor necrosis factor (TNF-a), interleukin-6 (IL-6), and interleukin-1 beta (IL-1ß) were considerably higher in the PFOS-exposed group. These inflammatory markers were significantly downregulated in the (PFOS + Que) treated group compared to the PFOS-exposed group (p 0.001), while these parameters still significantly increased compared to controls. rats treated with the extract (Fig. 5).

### Cardiac p53, Bax, Bcl-2, caspase-3, and SERCA2a mRNA expression in the different studied groups

**Fig. 6:**
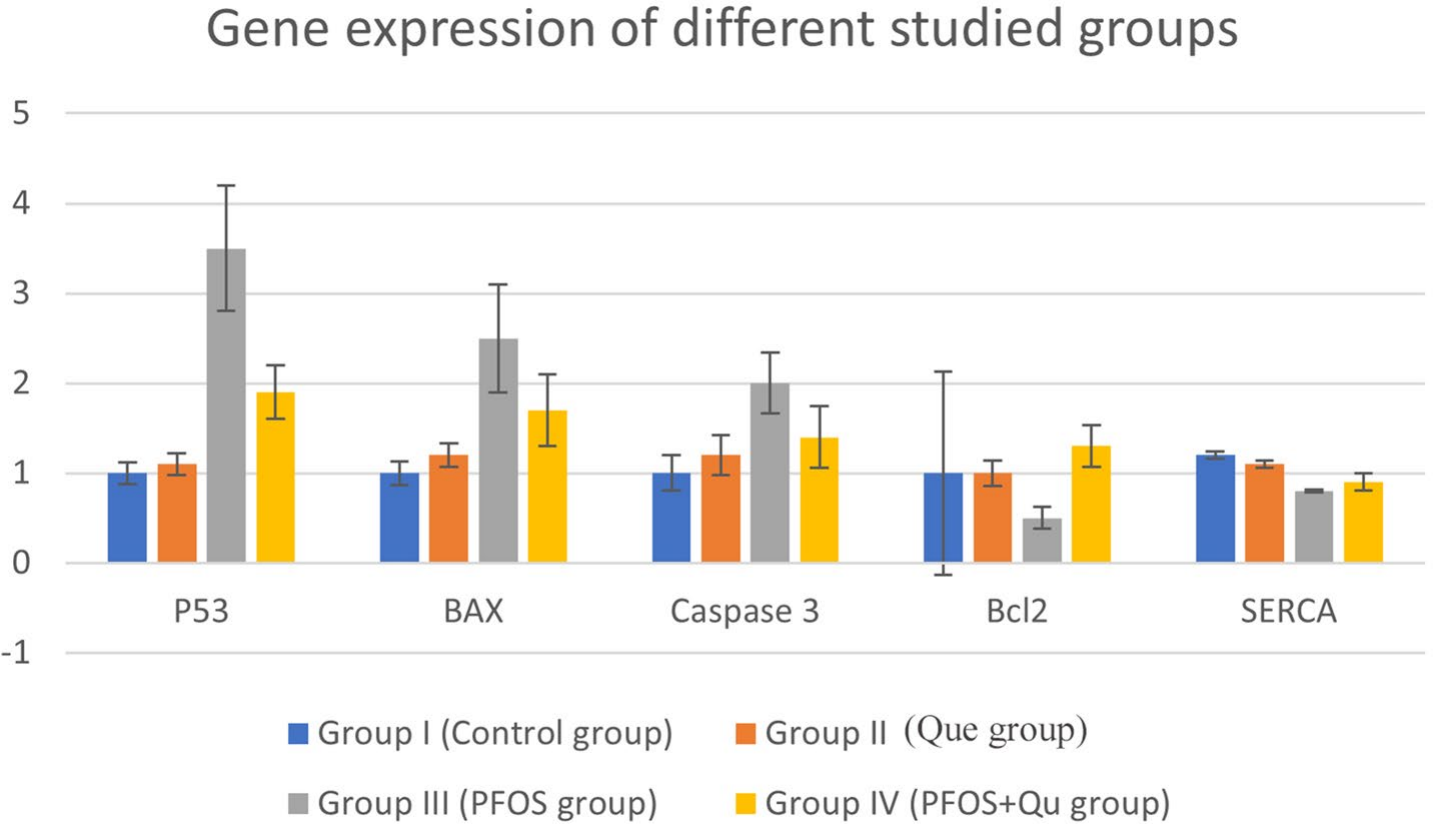
Bar charts representing the gene expression of different studied groups

The toxic effect of PFOS administration showed apoptotic effects on cardiac tissues by increasing Bax, p53, and caspase 3 mRNA expression and decreasing Bcl2 mRNA expression compared to controls. SERCA2a mRNA expression was significantly decreased in the PFOS-treated group compared to controls. When compared to the PFOS group, QU (PFOS + Que) had anti-apoptotic effects, as evidenced by a significant decrease in Bax, p53, and caspase 3 mRNA expression and an increase in Bcl2 mRNA expression. However, there was no discernible influence on SERCA2a expression. Group II showed no significant change compared to the control group (Fig. 6).

## Discussion

According to previous research, PFOS is a persistent organic pollutant with a bioaccumulation effect, making it a significant health risk (Tsuda 2016). PFOS has negative impacts on different organs, with the heart having the second highest PFOS bioaccumulation behind the liver (Yang et al. 2020). To our knowledge, few investigations on PFOS' cardiovascular toxicity have been published, and its cardiotoxicity is still unknown. Different underlying mechanisms for PFOS cardiotoxicity are investigated in this research. In this research, PFOS exposure caused oxidative stress, as evidenced by considerably higher MDA levels and lower antioxidant enzymes GSH, GPX, and GSH. In microvascular endothelial cells, exposure to 25 or 50 mg/L PFOS for one hour resulted in the formation of ROS, actin filament remodeling, and changes in endothelial permeability (Qian et al. 2010). The actin filament remodeling that contributed to increased endothelial permeability was regulated by PFOS-induced ROS, although the regulatory mechanism is unknown.

The synthesis of reactive metabolites causes mitochondrial oxidative stress, which is linked to changes in calcium homeostasis. The mitochondrial permeability transition (MPT) pores open as a result. This cascade also includes the release of an apoptosis-inducing factor (Hinson et al. 2010; Jaeschke et al. 2012).

We studied the molecular mechanisms of apoptosis by assessing the expression of pro-apoptotic and anti-apoptotic genes to see if PFOS had any effect on apoptotic pathways. When compared to controls, the apoptotic impact of PFOS was validated by increased cardiac tissue expression of Bax, p53, and caspase 3 mRNA and decreased cardiac tissue Bcl2 mRNA expression. In line with our findings, (Zeng et al. 2015) discovered that PFOS exposure causes cardiomyocyte apoptosis in rats using tunnel analysis. This was corroborated by a histological study of the heart tissue in the PFOS group, which revealed darkly pigmented pyknotic nuclei in the cardiomyocytes. The apoptotic process, which appears to play a key role in PFOS toxicity, could explain this conclusion (Xu et al. 2020). PFOS can cause apoptosis in murine N9 cells and upregulate the number of apoptotic cells in the liver of adult rats, according to Zhang et al.2011, which is consistent with our findings.

Increased collagen fibers and fibroblast aggregation induced by PFOS could imply that oxidative stress and PFOS consumption are key contributors to cardiac fibrosis and endothelial dysfunction. Parallel with our results, previous research (Xu et al. 2022) stated that in rats, 10 mg/kg PFOS caused considerable cardiac fibrosis and myocardial enlargement, as well as elevated biochemical indices supporting myocardial injury. This was proven statistically as a substantial increase in the mean area percent of collagen fibers in the PFOS group when compared to the control group, whereas Que was able to show a significant decrease in this mean area percent when compared to the PFOS group.

A previous study (Wang et al. 2021) proposed that the molecular mechanism ruling out PFOS-induced inflammation is mainly mitochondrial dysfunction and the release of mtDNA. TNF and Interleukin-6 (IL-6) levels were measured in our study to assess the inflammatory effects of PFOS on cardiotoxicity. According to our findings, TNF-a, IL-6, and Interleukin-1 beta (IL-1ß) levels were significantly higher in the PFOS exposed group, as were inflammatory cellular infiltrates found on histological inspection of the PFOS exposed group (Sunderland et al. 2019) this was parallel with our results, which asserted, that PFOS deposited in diverse tissues is ingested by local macrophages, resulting in the release of multiple proinflammatory cytokines (such as IL-1, IL-6, and TNF-), resulting in cell damage and inflammation.

Thyroid structure and function can potentially be harmed by PFOS exposure. T3 and T4 levels in the PFOS-exposed group were significantly lower than in the control group (Coperchini et al. 2015) Normally, nearby heart cells communicate with one another via cardiac gap junctions, which are channels that connect cardiomyocytes and provide the foundation of the heart's electrical communication network. It is mostly made up of a protein called connexin-43 (Dhein et al. 2002). Connexin-43 (Cx43) is a significant cardiac connexin protein that is expressed in the intercalated discs of cardiomyocytes in the myocardium of adult rats (Severs et al. 2008) in the nucleus (Dang et al. 2003) and in mitochondria and play an essential role in the propagation of action potential, metabolic coupling, development of the heart, and tissue homeostasis.

In the present study, the PFOS group exhibited weak immunoexpression of CX-43 in the intercalated discs, which displayed faintly stained fragmented-bands in comparison with the control group. Que treatment, on the other hand, improved impaired Cx43 expression and restored normal cardiac function in group IV, indicating its cardioprotective action. Such a reduction in the expression of Cx43 is a prevalent finding in chronic heart diseases, such as ischemia, myocarditis, idiopathic cardiomyopathies, and heart failure (Roell et al. 2007) Another study (Pecoraro et al. 2017) hypothesize that the reduced expression of Cx43 in the heart is linked to calcium overload and alterations in cardiac functions.

The heart is one of the primary targets of thyroid hormone (TH) action, and its functions may be disrupted because of the impairment of thyroid hormone signaling caused by PFOS exposure; in fact, any change in TH impacts cardiac metabolism directly or indirectly (Ho et al. 2013; Tsutsui et al. 2011).

We evaluated the in vitro effect of PFOS on thyroid cells and found that at a PFOS concentration of 100 M (50 mg/L), there was obvious cytotoxicity (inhibited cell proliferation and increased cell death). The authors looked into how PFOS got into thyroid cells and discovered that it did so through a passive diffusion process. Through hydrophobic contact and hydrogen bonding, PFOS can directly connect to the T3 receptor (Ren et al. 2015).

Intracellular free Ca2 + concentration, which is regulated by a synergistic interaction between Ca2 + release from the sarcoplasmic reticulum through ryanodine receptors and Ca2 + re-uptake through the sarcoplasmic reticulum calcium-activated ATPase (SERCA2), is a factor regulating cardiac muscle contraction/relaxation activity(Kushnir and Marks 2010).

The alterations in thyroid hormone signaling are associated with cardiac pathophysiology (Galli et al. 2010). In our study, we evaluated the alteration of thyroid hormone occurring in the PFOS group on heart tissue by measuring the cardiac mRNA expression of SERCA2 levels. T3 positive activation of SERCA2 determines accelerated sarcoplasmic reticulum Ca2 + re-uptake (Kahaly and Dillmann 2005). The hypothyroid condition is characterized by an impairment of Ca2 + intracellular loading and improvement of systolic function (Mastorci et al. 2020). Our result showed a significant decrease in SERCA2 expression in the PFOS exposed group, suggesting a negative inotropic effect of PFOS exerting its effect through hypothyroidism.

In our daily diet, Qu is the most common and abundantly dispersed flavanol component. It can be found in practically all plant foods, including tea, onions, lettuce, broccoli, beans, fruits, and buckwheat, as well as gingko leaves, mulberry berries, sandalwood, and other Chinese herbs (David et al. 2016; Frankel et al. 1993).

Que was chosen in this study because of its numerous biological targets, including inhibiting the formation of reactive oxygen species by blocking nicotinamide-adenine dinucleotide phosphate (NADPH) oxidase (Xiao et al. 2017), preventing the formation of atherosclerotic plaques by upregulating nitric oxide synthetase (Calabró et al. 2018), and downregulating matrix metalloproteinase-1 (MMP-1) helps stabilize endothelium atherosclerotic plaques, which helps protect against CVDs (Xiao et al. 2017).

In the current study, Que enhanced heart function, as shown by significant reductions in cardiac enzymes, serum total cholesterol, LDLC, and triglyceride levels, MDA levels, and (TNF-a), Interleukin-6 (IL-6), and Interleukin-1 beta (IL-1ß) levels, Thyroid hormones, CAT, and SOD levels were also improved. It improves heart morphology significantly. Previous clinical research (Kosari-Nasab et al. 2019), which stated Que's cardioprotective effects, backed this up.

Furthermore, another study found that individuals with considerable levels of inflammation and oxidative stress benefited more from quercetin administration, which is consistent with our findings (Boots et al. 2011). On the other hand, healthy people have lower levels of oxidative stress and cytokine. In cell cultures, quercetin also suppressed the synthesis of the inflammatory cytokine TNF-. This effect is linked to changes in the NF-kB pathway (Boots et al. 2008).

In a cross-sectional study of lipid profiles, quercetin consumption was found to be inversely associated to low-density lipoprotein cholesterol (LDL-C) (Arai et al. 2000). Only one study from 2009 found that quercetin ingestion reduced HDL-C levels significantly when compared to placebo (Egert et al. 2009) which was in line with our findings.

## Conclusion

In summary, the current study found that PFOS exposure caused pathological changes in adult rats’ hearts, including cardiac fibrosis and myocardial hypertrophy, which was possibly linked to an increase in apoptosis, as evidenced by increased Bax, p53, and caspase 3 mRNA expression and decreased Bcl2 mRNA expression compared to controls, as well as proinflammatory cytokines such as TNF-a, IL-6. Furthermore, the related cardiac pathophysiology may be explained by changes in thyroid hormone signaling caused by PFOS exposure.

This study found that quercetin consumption improved not just the histopathological effects of PFOS, but also the biochemical and molecular consequences.
